# Analysis of the interferon-γ-induced secretome of intestinal endothelial cells: putative impact on epithelial barrier dysfunction in IBD

**DOI:** 10.3389/fcell.2023.1213383

**Published:** 2023-08-14

**Authors:** Elisabeth Naschberger, Christian Flierl, Jinghao Huang, Lena Erkert, Reyes Gamez-Belmonte, Miguel Gonzalez-Acera, Magdalena Bober, Martin Mehnert, Christoph Becker, Vera S. Schellerer, Nathalie Britzen-Laurent, Michael Stürzl

**Affiliations:** ^1^ Division of Molecular and Experimental Surgery, Department of Surgery, Universitätsklinikum Erlangen, Friedrich-Alexander-Universität Erlangen-Nürnberg (FAU), Erlangen, Germany; ^2^ Department of Medicine I, Universitätsklinikum Erlangen, Friedrich-Alexander-Universität Erlangen-Nürnberg (FAU), Erlangen, Germany; ^3^ Biognosys AG, Schlieren, Switzerland; ^4^ Department of Pediatric Surgery, University Medicine Greifswald, Greifswald, Germany; ^5^ Division of Surgical Research, Department of Surgery, Universitätsklinikum Erlangen, Friedrich-Alexander-Universität Erlangen-Nürnberg (FAU), Erlangen, Germany; ^6^ Comprehensive Cancer Center Erlangen-EMN, Universitätsklinikum Erlangen, Erlangen, Germany

**Keywords:** IBD-inflammatory bowel disease, cytokines, interferon, secretion, angiocrine, paracrine, barrier, endothelial

## Abstract

The development of inflammatory bowel diseases (IBD) involves the breakdown of two barriers: the epithelial barrier and the gut-vascular barrier (GVB). The destabilization of each barrier can promote initiation and progression of the disease. Interestingly, first evidence is available that both barriers are communicating through secreted factors that may accordingly serve as targets for therapeutic modulation of barrier functions. Interferon (IFN)-γ is among the major pathogenesis factors in IBD and can severely impair both barriers. In order to identify factors transmitting signals from the GVB to the epithelial cell barrier, we analyzed the secretome of IFN-γ-treated human intestinal endothelial cells (HIEC). To this goal, HIEC were isolated in high purity from normal colon tissues. HIEC were either untreated or stimulated with IFN-γ (10 U/mL). After 48 h, conditioned media (CM) were harvested and subjected to comparative hyper reaction monitoring mass spectrometry (HRM™ MS). In total, 1,084 human proteins were detected in the HIEC-CM. Among these, 43 proteins were present in significantly different concentrations between the CM of IFN-γ- and control-stimulated HIEC. Several of these proteins were also differentially expressed in various murine colitis models as compared to healthy animals supporting the relevance of these proteins secreted by inflammatory activated HIEC in the inter-barrier communication in IBD. The angiocrine pathogenic impact of these differentially secreted HIEC proteins on the epithelial cell barrier and their perspectives as targets to treat IBD by modulation of trans-barrier communication is discussed in detail.

## Introduction

Inflammatory bowel diseases (IBD) affect several million individuals worldwide, with Crohn’s disease (CD) and ulcerative colitis (UC) being the clinically predominant forms. IBD similarities are based on their common presentation as intestinal chronic inflammatory disorders characterized by cyclic flares of destructive inflammation resulting in severe impact on the intestinal barrier functions (Zhang and Li, 2014). Heterogeneity is present at the levels of clinical presentation, immune reactions, molecular-genetic components and microbial players involved (Lloyd-Price et al., 2019).

The intestinal barrier serves manifold tasks, which is also evident from its complex structure composed of two sequential physical barriers. The first barrier from the intestinal lumen is established by the epithelial barrier that consists of a single cell layer of epithelial cells overlaid by a mucus layer, which physically separates the microbiota in the gut lumen from epithelial cells (Stürzl et al., 2021). Directly below this epithelial barrier lies the gut-vascular barrier (GVB) controlling the entry of molecules and cells into the portal circulation and their subsequent delivery to the liver (Spadoni et al., 2015; Spadoni et al., 2017).

The structure and functions of the epithelial barrier have been comprehensively described in previous work (López-Posadas et al., 2017). In contrast, the existence and significant contribution of the GVB to IBD has been recognized only recently. Clinical evidence for a role of the GVB in IBD was obtained by the observation that the vasculature in patients exhibits increased permeability during acute phases of the disease, which is decreasing or absent in remission phases (Langer et al., 2019). In addition, studies in preclinical mouse models revealed that a breakdown of the GVB in the colon allows the permeation of bacteria into the blood with access to distant organs, including the liver, with significant impact on IBD pathogenesis (Spadoni et al., 2015). In own studies, we detected that IFN-γ, an immune-modulatory cytokine with driver activity in IBD pathogenesis, increases vascular permeability in the dextran sodium sulfate (DSS)-induced colitis model (Langer et al., 2019). Increased intestinal blood vessel permeability was associated with structural and functional perturbations of the adherens junction protein vascular endothelial (VE)-cadherin and significant worsening of the disease. An endothelial specific knock-out of the IFN-γ-receptor 2 (IFNγR2) as well as pharmacological vessel stabilization in mouse models suppressed vascular permeability and the development of acute and chronic DSS-colitis (Langer et al., 2019). These results provided clear evidence for the importance of the vascular barrier in IBD.

Effective cooperation of two different barriers requires coordinated action and communication. Well-established communication pathways between the epithelial barrier and the GVB are indicated by the observation that nutrient composition in the gut can affect the blood flow (Stan et al., 2012; Gentile and King, 2018). In addition, epithelial cells can secrete factors in response to pathogens such as cytokines, chemokines, reactive oxygen species, and lipid mediators, which can activate endothelial cells (Boueiz and Hassoun, 2009; Franze et al., 2016; Ferrari et al., 2017; Gentile and King, 2018).

However, the endothelium is not only a passive tube system transporting blood and receiving signals from surrounding cells, but exerts perfusion-independent functions, which actively contribute to the tissue microenvironment in organ development and diseases. In IBD, the intestinal microvasculature is notably involved in immune cell recruitment through expression of cell adhesion molecules (CAMs), such as VCAM1 or MadCAM1 (Binion et al., 1998). The inhibition of T-cell recruitment by targeting the binding of α4β7 integrins to endothelial MadCAM1 represents a new therapeutic axis in IBD (Neurath, 2017).

The first hint for an active paracrine function of the endothelium within the tissue microenvironment was derived from cancer research (Butler et al., 2010). Subsequent studies identified tumor repressive molecules that are expressed and released from endothelial cells, including the slit homolog 2 protein (Slit 2), perlecan, thrombospondin and SPARCL1 (Butler et al., 2010; Franses et al., 2011; Naschberger et al., 2016; Hinshaw and Shevde, 2019). Now, it is generally accepted that endothelial cells can actively trigger the microenvironment via so called “angiocrine factors” -a term that. includes secreted and membrane-bound inhibitory or stimulatory growth factors, trophogens, chemokines, cytokines, extracellular matrix components, exosomes, and other cellular products expressed by endothelial cells (Rafii et al., 2016).

Angiocrine functions in IBD have not been extensively investigated as yet. Only recently, we performed a meta-analysis to investigate whether angiocrine signaling in the colon may impact epithelial barrier functions (Stürzl et al., 2021). This approach yielded six putative candidates that are secreted from endothelial cells and may contribute to IBD pathogenesis, including proteins of the von Willebrand factor domain superfamily (VWA1, vWF), tissue inhibitor of metalloproteinases (TIMP)-1, matrix metalloproteinase (MMP)-14, the chemokine CXCL10, and the matricellular protein SPARCL1 (Stürzl et al., 2021). The expression and known functions of these proteins supported the hypothesis that they may be active in IBD. However, the bioinformatical analysis also showed that the overlap of genes retrieved from the different studies was very low, which was well in agreement with the high variation of activation and organ-dependent plasticity of endothelial cells (Stürzl et al., 2021).

## Analysis of the IFN-γ-induced secretome in HIEC

**FIGURE 1:**
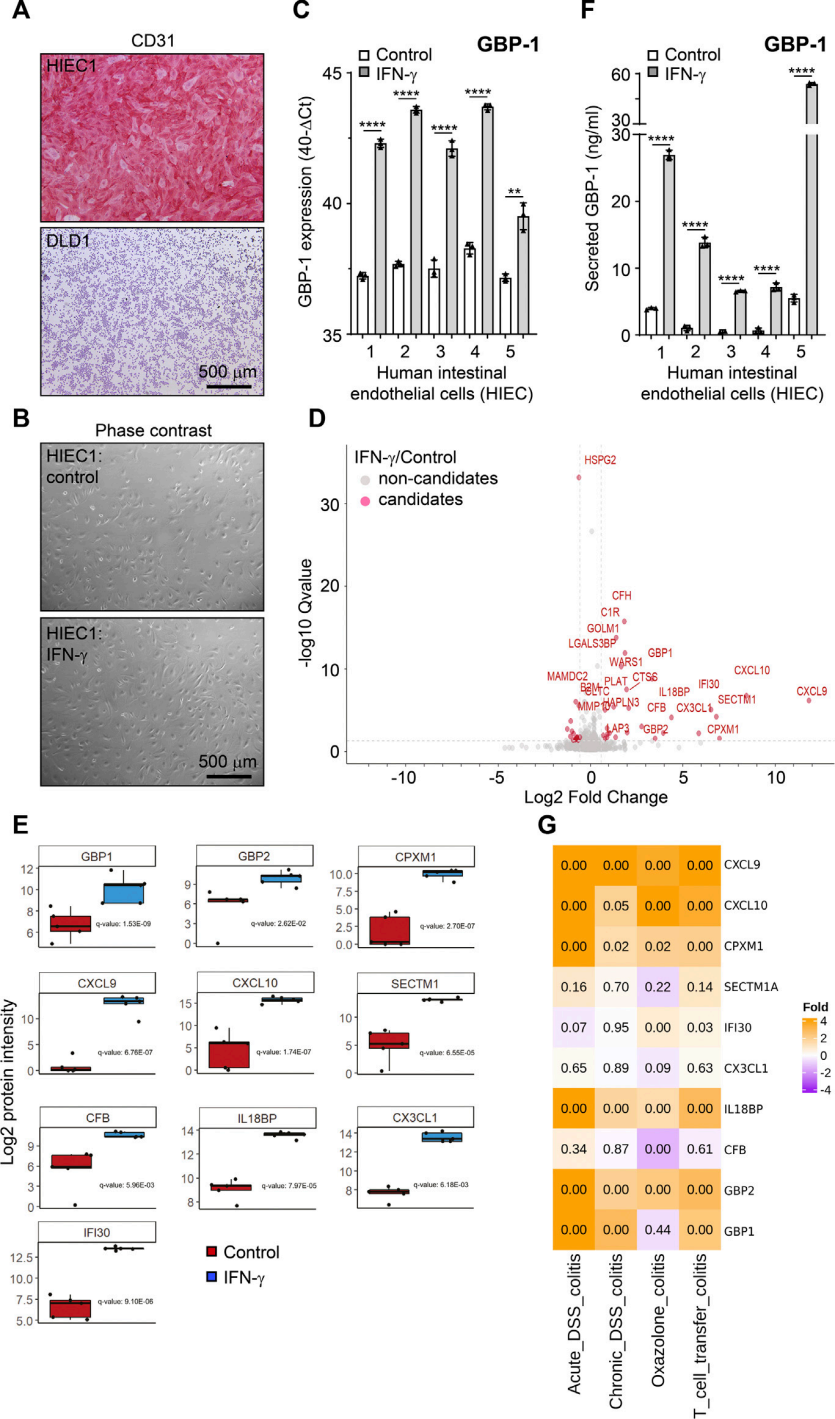
The secretome of IFN-γ-treated human intestinal endothelial cells. **(A)** Cultivated human intestinal endothelial cells (HIEC) uniformly express the endothelial cell-specific CD31 antigen whereas the epithelial colorectal cancer cell line DLD1 is negative. **(B)** No difference in the cell phenotype is detected in untreated and IFN-γ-treated HIEC. **(C)** IFN-γ treatment (10 U/mL, 48 h) induces expression of GBP-1 in all HIEC cultures as determined by RT-qPCR. **(D)** Volcano blot of the secretome of IFN-γ-treated HIEC. Proteins present in significantly different concentrations in the cell culture supernatants of IFN-γ-treated and untreated HIEC are indicated in red. **(E)** Box blots showing differential secretion of the different factors in all HIEC cultures (n = 5) in response to IFN-γ. *p*-values were calculated with the one sample *t*-test (μ = 0) and were corrected for overall FDR using the q-value approach (Storey and Tibshirani, 2003). **(F)** IFN-γ treatment (10 U/mL, 48 h) induces secretion of GBP-1 in all HIEC cultures as determined by GBP-1-specific ELISA. **(G)** Expression of genes encoding the top ten secreted proteins from IFN-γ-treated HIEC in different experimentally induced murine colitis models. Expression relative to healthy control mice is indicated by color code. Numbers are representing adjusted *p*-values of statistical differences. **(A,B)** Scale bars correspond to 500 µm. **(C,F)** *p* values: *** = *p* <0.001, ** = *p* <0.01, and * = *p* <0.05, paired *t*-test.

Here we aimed to determine putative angiocrine factors released from cultivated primary human intestinal endothelial cells (HIEC) under pathogenically relevant stimulation in an experimental approach. Based on own previous results we used IFN-γ as a model cytokine for stimulation (Langer et al., 2019). In order to reduce pathogenesis-related heterogeneity we refrained from using patient-derived human HIEC but focused on highly pure cultures of healthy HIEC instead. To this goal, HIEC were isolated from healthy colon areas of five patients who underwent surgical therapy for colorectal cancer (CRC) (see Supplementary Methods). Endothelial cells were isolated by FACS-based cell sorting following previously established protocols (Naschberger et al., 2016; Naschberger et al., 2018). A purity above 98% of all five cultures was determined with reverse transcription quantitative polymerase chain reaction (RT-qPCR) and cytochemistry as described previously (Naschberger et al., 2016; Naschberger et al., 2018) and is exemplarily shown here by the uniform expression of the endothelial marker CD31 in all HIEC of the different patients but not in the CRC tumor cell line DLD1 (Figure 1A, Supplementary Figures S1A).

The five HIEC cultures were treated with IFN-γ which did not induce different morphology in untreated as compared to treated HIEC (Figure 1B, quantitative evaluation Supplementary Figures S1B). However, successful stimulation of all five cultures was indicated by the expression of IFN-γ-induced guanylate binding protein-1 (GBP-1), a well-established marker for IFN-γ stimulation of eukaryotic cells (Guenzi et al., 2001; Lubeseder-Martellato et al., 2002), which was highly increased in all stimulated HIEC (Figure 1C).

Next, cell culture supernatants were harvested from the IFN-γ-treated and untreated HIEC and subjected to hyper reaction monitoring mass spectrometry (HRM™ MS). Comparison of stimulated and unstimulated cultures identified 1,713 proteins detected by a mean of 5.79 peptides per protein. From all proteins identified, 1,084 were of human origin (629 from medium FBS) with 43 proteins differentially secreted between the CM of IFN-γ-stimulated and unstimulated HIEC (Figure 1D, red; Table 1). The top ten differentially secreted proteins included the chemokines CXCL9, CXCL10 and fractalkine as well as the IFN-γ-induced secreted proteins secreted and transmembrane protein 1 (SECTM1 or K12), gamma-interferon-inducible lysosomal thiol reductase (IFI30) and GBP-1 (Figure 1E). Of note, increased secretion of GBP-1 by IFN-γ-treated HIECs as detected by mass spectrometry was confirmed by independent ELISA (Figure 1F).

**TABLE 1:** Significantly changed proteins between the supernatants of IFN-γ treated and untreated HIEC.

Protein description	Gene ID	Number of precursors	Ratio	*p*-value
C-X-C motif chemokine 9	CXCL9	3	3625.19	8.43E-09
C-X-C motif chemokine 10	CXCL10	8	348.02	2.00E-09
Probable carboxypeptidase X1	CPXM1	1	126.95	3.15E-03
Secreted and transmembrane protein 1	SECTM1	4	112.59	1.70E-06
Gamma-interferon-inducible lysosomal thiol reductase	IFI30	5	92.04	1.89E-07
Fractalkine	CX3CL1	2	58.11	3.79E-04
Interleukin-18-binding protein	IL18BP	3	20.93	2.32E-06
Complement factor B	CFB	2	15.52	3.60E-04
Guanylate-binding protein 2	GBP2	3	11.41	2.94E-03
Guanylate-binding protein 1	GBP1	14	10.09	1.27E-11
Hyaluronan and proteoglycan link protein 3	HAPLN3	7	6.86	3.88E-05
Cathepsin S	CTSS	11	4.23	1.13E-07
Signal transducer and activator of transcription 1-alpha/beta	STAT1	3	4.00	2.74E-04
Tryptophan-tRNA ligase. cytoplasmic	WARS1	12	3.91	2.85E-10
Golgi membrane protein 1	GOLM1	16	3.67	6.27E-15
Complement factor H	CFH	31	3.57	5.47E-19
Galectin-3-binding protein	LGALS3BP	15	3.20	3.72E-13
Complement C1r subcomponent	C1R	11	2.65	6.89E-17
Cytosol aminopeptidase	LAP3	3	2.58	1.86E-03
Tissue-type plasminogen activator	PLAT	20	2.41	7.70E-08
Legumain	LGMN	11	2.07	3.38E-04
HLA class I histocompatibility antigen. C alpha chain	HLA-C	13	1.91	5.51E-04
Cystatin-C	CST3	10	1.88	1.88E-03
Keratin. type I cytoskeletal 14	KRT14	10	1.88	6.29E-05
Midkine	MDK	8	1.74	3.10E-03
Beta-2-microglobulin	B2M	5	1.72	2.06E-07
Procathepsin L	CTSL	7	1.61	8.39E-04
Glypican-1	GPC1	7	0.67	1.87E-03
Basement membrane-specific heparan sulfate proteoglycan core protein	HSPG2	170	0.64	7.03E-37
Clathrin heavy chain 1	CLTC	30	0.64	5.30E-08
X-ray repair cross-complementing protein 6	XRCC6	6	0.63	5.72E-03
Endothelial cell-specific molecule 1	ESM1	6	0.60	1.67E-03
Ephrin type-B receptor 4	EPHB4	2	0.60	1.71E-03
Ephrin-A1	EFNA1	4	0.59	2.42E-03
MAM domain-containing protein 2	MAMDC2	20	0.58	1.55E-08
Eukaryotic translation initiation factor 3 subunit B	EIF3B	4	0.57	4.86E-03
Vesicle-trafficking protein SEC22b	SEC22B	2	0.57	4.10E-03
Lymphatic vessel endothelial hyaluronic acid receptor 1	LYVE1	4	0.53	4.67E-04
Splicing factor. proline- and glutamine-rich	SFPQ	4	0.52	6.29E-03
Annexin A6	ANXA6	6	0.50	1.91E-04
Stromelysin-2	MMP10	9	0.48	7.77E-06
Placenta growth factor	PGF	3	0.47	1.22E-03
Protein SETSIP; Protein SET	SETSIP, SET	2	0.42	8.19E-05

Interestingly, with the exception of CXCL10 no further top candidate of our previous meta-analysis was detected in the present study confirming that endothelial cells exhibit high tissue-related heterogeneity and suggesting that HIEC should be preferentially used in order to obtain data of relevance for IBD.

## Pathogenic impact of the vascular IFN-γ-secretome in IBD

In order to determine the pathogenic impact of the intestinal vascular IFN-γ-induced secretome, the expression of the top ten secreted proteins was examined in different murine models of experimentally induced colitis, including acute and chronic DSS-colitis as well as oxazolone-induced colitis and T-cell transfer colitis in a next step. The expression of the genes encoding CXCL9, CXCL10, CPMX1, IL18BP and GBP2 was highly significantly increased in each of the different models (Figure 1G). Moreover, GBP-1 and IFI30 showed an increased expression in three and two of the models, respectively (Figure 1G). Only SECTM1, fractalkine and CFB did not show a significant increase of expression in any of the different colitis models (Figure 1G). Altogether, seven of the ten genes, encoding for the most differentially secreted proteins from HIEC in the presence of IFN-γ, also showed significantly increased expression in experimentally induced colitis models, supporting their function in pathogenesis. In the following, the most relevant top candidates involved in IBD pathogenesis retrieved by our screening are discussed in detail.

## Discussion

### CXCL10

CXCL10 is an 8.7 kDa non-glutamic acid leucine-arginine (ELR)-CXC chemokine, which acts as a ligand for the CXCR3 receptor (Singh et al., 2007). CXCL10 is secreted by several cell types, including endothelial cells, in response to IFN-γ to induce the recruitment and activation of CXCR3+ cells (Singh et al., 2007). CXCL10 is upregulated in colonic tissues of patients with UC and CD compared to control non-IBD tissues (Uguccioni et al., 1999; Zahn et al., 2009; Schroepf et al., 2010; Hosomi et al., 2011; Ostvik et al., 2013). Accordingly, the number of CXCR3-expressing immune cells is increased in the lamina propria of IBD patients (Singh et al., 2007). Expression of CXCL10 in colon biopsies correlates with secondary loss of response to anti-TNF-α therapy after achieving an initial response (Luther et al., 2018). Elevated CXCL10 serum levels correlate with extra-intestinal manifestations indicating that CXCL10 is released into the circulation during IBD (Martinez-Fierro et al., 2019). Furthermore, CXCL10 serum levels are increased in IBD patients with unstable remission compared to patients with stable remission (Kessel et al., 2021). Based on these findings, several clinical trials were performed to test the efficacy of eldelumab, a human monoclonal antibody against CXCL10, as treatment for UC (Trivedi and Adams, 2018). Despite trends towards clinical response and remission, the primary and secondary end points were not met and further dose-response or combination studies are warranted (Danese and Panés, 2014; Mayer et al., 2014; Sandborn et al., 2016). In murine colitis models, inhibition of CXCL10 reduces intestinal inflammation (Sasaki et al., 2002; Singh et al., 2003; Hyun et al., 2005; Suzuki et al., 2007; Zhao et al., 2017) but also had unexpected effects on intestinal epithelial cells (Sasaki et al., 2002; Singh et al., 2003; Hyun et al., 2005; Suzuki et al., 2007; Zhao et al., 2017). Neutralization of CXCL10 resulted in increased epithelial cell proliferation and decreased apoptosis, which resulted in reduced epithelial ulceration and longer colon crypts (Sasaki et al., 2002; Suzuki et al., 2007). In addition, CD patients with the highest levels of the IFN-γ-induced chemokines CXCL9, CXCL10 and CXCL11 showed hypertrophied epithelial layers at multiple sites (Singh et al., 2007). These findings suggest that CXCL10 secreted by endothelial cells during intestinal inflammation is not only involved in immune cell recruitment but also crypt cell growth regulation and extra-intestinal manifestations.

### CXCL9

Similarly to CXCL10, CXCL9 is a CXC-chemokine induced by IFN-γ in numerous cell types. It also binds to the CXCR3 receptor, and is involved in the recruitment of granulocytes and mononuclear cells. CXCL9 expression is increased in mucosal samples of UC and CD patients (Hosomi et al., 2011; Elia and Guglielmi, 2018; Caruso, 2019) and positively correlates with disease activity and negatively with response to treatment using corticosteroids in UC or anti-TNF-α in CD (Egesten et al., 2007; Lacher et al., 2007; Luther et al., 2018; Zhong et al., 2022). Serum CXCL9 levels also reflect disease activity in both UC and CD (Caruso, 2019; Bergemalm et al., 2021; Boucher et al., 2022; Chen et al., 2022) and circulating CXCL9 was identified in preclinical CD and UC as an IBD-risk biomarker (Bergemalm et al., 2021; Leibovitzh et al., 2023) that predicts relapse in UC and CD (Kessel et al., 2021; Walshe et al., 2022). At the molecular level, CXCL9 has been shown to inhibit the reconstitution of the intestinal mucosa after injury (Lu et al., 2015) and to control *E. coli* overgrowth through the pyruvate dehydrogenase-encoding *aceE* gene in a DSS-induced colitis model (Wei et al., 2022). Hence, CXCL9 released by endothelial cells might not only increase immune cell recruitment but also may compromise the epithelial barrier and alter the microbiota in intestinal inflammation.

### Fractalkine/CX3CL1

Fractalkine (FKN/CX3CL1) is a transmembrane protein which mediates leukocyte adhesion to endothelial cells (Sans et al., 2007). In addition, a soluble form of fractalkine with chemoattractive properties is secreted by cleavage. Its receptor, CX3CR1, is expressed primarily on the surface of monocytes, natural killer cells, and CD8^+^ T cells and mediates both adhesive and chemoattractive functions (Sans et al., 2007). Fractalkine expression is upregulated by inflammatory cytokines (IFN-γ, IL-1β and TNF-α) or by direct leukocyte contact (Muehlhoefer et al., 2000; Sans et al., 2007), and has been detected in intestinal epithelial cells and endothelial cells both in normal small intestine and in active Crohn’s disease mucosa (Muehlhoefer et al., 2000). However, significantly higher levels of fractalkine mRNA were found in the intestine during active CD and UC (Muehlhoefer et al., 2000; Brand et al., 2006; Kobayashi et al., 2007). Similarly, HIECs isolated from IBD patients exhibited significantly stronger fractalkine expression as compared to control HIECs (Sans et al., 2007). This correlated with significantly higher numbers of mucosal circulating CX3CR1+ T cells in active IBD compared to inactive IBD or healthy subjects (Kobayashi et al., 2007; Sans et al., 2007). The presence of two *CX3CR1* polymorphisms (T280M and V249I) has been associated with intestinal stenosis in CD patients (Brand et al., 2006; Sabate et al., 2008). The knockout/blockade of fractalkine attenuated mucosal inflammation in murine colitis models and showed a moderate clinical response in CD patients (Wakita et al., 2017; Kuboi et al., 2019; Tabuchi et al., 2019; Matsuoka et al., 2021). Targeting endothelial fractalkine might be particularly important to block leukocyte adhesion and migration, platelet adhesion and even angiogenesis (Scaldaferri et al., 2009; Rutella et al., 2011).

### GBP-1 and GBP-2

Two members of the guanylate binding protein family, GBP-1 and GBP-2, were detected in our analysis. GBPs are large GTPases, which are expressed in response to stimulation by inflammatory cytokines (Britzen-Laurent et al., 2016). GBP-1 is among the most highly induced proteins by IFN-γ in eukaryotic cells. *In vivo*, a strong expression of GBP-1 is associated with the presence of inflammation and was detected in inflamed tissues during autoimmune diseases or IBD, where it is mostly associated with blood vessels (Lubeseder-Martellato et al., 2002; Haep et al., 2015; Ning et al., 2023). In pediatric patients with IBD, a high expression of GBP-1 was associated with an absence of early response to anti-TNF treatment (Salvador-Martín et al., 2021). Murine GBP-1/GBP-2b is also upregulated during experimental colitis (de Buhr et al., 2006). Intracellular expression of GBP-1 inhibits angiogenesis in endothelial cells (Guenzi et al., 2001), and inhibits proliferation and migration in tumor cells and intestinal epithelial cells, while preventing cell apoptosis (Schnoor et al., 2009; Britzen-Laurent et al., 2013; Ostler et al., 2014). GBP-1 is also able to regulate T-cell receptor signaling (Forster et al., 2014). Interestingly, GBP-1 is specifically and efficiently secreted from endothelial cells by a non-classical, likely ABC transporter-dependent, pathway (Naschberger et al., 2006; Naschberger et al., 2017; Carbotti et al., 2020). GBP-1 has been detected in the serum or cerebrospinal fluid during infectious and inflammatory diseases including bacterial meningitis, systemic lupus erythematosus, rheumatoid arthritis and systemic sclerosis (Naschberger et al., 2006; Hammon et al., 2011; Naschberger et al., 2017). The functions of secreted GBP-1 and GBP-2 remain unknown and further studies are warranted to investigate their potential as blood biomarkers in IBD, as well as their function on the intestinal epithelial barrier.

### IL-18BP

IL-18 binding protein (IL-18BP) is a natural circulating high-affinity antagonist of interleukin-18 (IL-18), which belongs to the IL-1 superfamily. While IL-18 is produced by a range of immune and non-immune cells including macrophages, dendritic cells (DCs), fibroblasts and intestinal epithelial cells, its receptor (IL-18R) is expressed by T cells, macrophages, NK-cells or endothelial cells (Kaplanski, 2018). IL-18BP blocks the binding of IL-18 to IL-18R, thereby dampening IFN-γ production. In children and adult CD patients, elevated expression of both IL-18 and IL-18BP has been detected in mucosal samples, with intestinal endothelial cells and macrophages being the major sources of IL-18BP (Corbaz et al., 2002). Higher IL-18 and IL-18BP levels have also been observed in the serum of IBD patients as compared to controls, which might be attributed to secretion by endothelial cells (Corbaz et al., 2002; Ludwiczek et al., 2005; Naftali et al., 2007; Leach et al., 2008). In particular in CD, circulating levels of both IL-18 and IL-18BP correlated with disease activity, which is well in agreement with the exacerbated Th1 immune response characteristic of the disease (Corbaz et al., 2002; Ludwiczek et al., 2005; Naftali et al., 2007; Leach et al., 2008). However, high levels of free unbound IL-18 are still detectable in CD patients, suggesting that IL-18BP is not produced in sufficient amounts to compensate the effects of IL-18 (Corbaz et al., 2002; Ludwiczek et al., 2005; Naftali et al., 2007; Leach et al., 2008). In DSS-induced experimental colitis the administration of IL-18BP or the neutralization of IL-18 was able to attenuate intestinal inflammation and weight loss (Siegmund et al., 2001; Sivakumar et al., 2002; Siegmund et al., 2004). IL-18BP may act anti-inflammatory not only by inhibition of immune cell recruitment but also through inhibition of IL-18-induced intestinal epithelial permeability (Allam et al., 2018). This is supported by the fact that, the knock-out of IL-18 in endothelial cells, hematopoietic cells or in intestinal epithelial cells was found to abrogate DSS-induced colitis, while the knock-out of IL-18R was only protective when present in intestinal epithelial cells (Nowarski et al., 2015). Overall, IL-18BP is produced and released during IBD, notably by endothelial cells, where it exerts protective effects by dampening the pro-inflammatory effects of IL-18.

### Complement factors

Our analysis has revealed an increased secretion of three complement system members in IFN-γ-stimulated intestinal endothelial cells: the complement C1r subcomponent (C1R) from the classical pathway and the complement factor B (CFB) and H (CFH) from the alternative pathway (Lubbers et al., 2017). Complement proteins are produced and secreted mostly by hepatocytes but also by endothelial cells, epithelial cells and leukocytes (Morgan and Gasque, 1997; Lubbers et al., 2017). IBD patients exhibit increased levels of circulating CFB (Nielsen et al., 1978; Campbell et al., 1982; Adinolfi and Lehner, 1988) and a similar increase of serum CFB has been observed in DSS-induced and bisphenol A (BPA)-induced experimental colitis in mice (Huang et al., 2022). More recently, genome-wide association studies (GWAS) have identified one SNP (rs4151657) at the CFB locus, which represents a risk variant for UC susceptibility (Juyal et al., 2015; Gupta et al., 2016; Shi et al., 2020; Mortlock et al., 2023). The presence of the rs4151651 SNP was associated with increased CFB expression, and CFB expression was shown to correlate with disease activity (Shi et al., 2020; Mortlock et al., 2023). CFB expression can be induced in human glomerular endothelial cells and intestinal epithelial cells by different inflammatory cytokines and is found in increased concentrations in the jejunal fluid of IBD patients (Ahrenstedt et al., 1990; Ostvik et al., 2014; Sartain et al., 2016). In contrast to CFB, very little is known about the role of CFH and C1r in IBD. C1r concentration was significantly increased in the serum of CD patients in clinical and serological remission in response to treatment with the anti-TNF-α antibody infliximab, suggesting an inverse correlation between C1r production and disease activity (Gazouli et al., 2013).

### Potential new angiocrine factors in IBD

Little is known about the role of CPXM1, IFI30 and SECTM1 in IBD, which were also among the top ten candidates of our screening. SECTM1 is an IFN-γ-regulated molecule acting as a co-stimulatory molecule in T and NK cells, where it binds CD7 (Lyman et al., 2000; Wang et al., 2012; Hubel et al., 2019). SECTM1 is expressed by antigen-presenting cells and epithelial cells that may also secrete a soluble form (Lam et al., 2005; Kamata et al., 2016). Carboxypeptidase X-1 (CPX-1), an inactive member of the metallocarboxypeptidase family encoded by the CPXM1 gene, is also a secreted protein (Reznik and Fricker, 2001; Kim et al., 2015). CPXM1 expression is upregulated in the inflamed intestinal mucosa of CD patients (Hong et al., 2017). Finally, IFN-γ-inducible lysosomal thiol reductase (IFI30/GILT) is a thiol reductase involved in the processing of antigenic proteins for antigen presentation by MHC class II molecules (Barjaktarević et al., 2006). Upregulation of IFI30 has been observed in uterine microvascular endothelial cells in response to IFN-γ (Kitaya et al., 2007).

In conclusion, the important pathogenic role of the vasculature in IBD has been appreciated only recently. Here, we identified and discussed factors secreted from HIEC in the presence of IFN-γ stimulation. Among these factors, CXCL9, CXCL10 and fractalkine have been already described to be closely associated with IBD pathogenesis either in preclinical murine models or in patients. Moreover, we identified novel factors secreted from IFN-γ-activated HIEC including GBP-1, GBP-2, CPXM1, IFI30 and SECTM1. These factors may warrant further studies on their role in IBD pathogenesis and as target for disease monitoring.

## Data Availability

Datasets are available on request: The raw data supporting the conclusions of this article will be made available by the authors, without undue reservation.
