# Supplementary figures and images for: Analysis of the interferon-γ-induced secretome of intestinal endothelial cells: putative impact on epithelial barrier dysfunction in IBD
Source: Front Cell Dev Biol. 2023 Aug 14;11:1213383. doi: 10.3389/fcell.2023.1213383 (PMC10460912; doi:10.3389/fcell.2023.1213383)

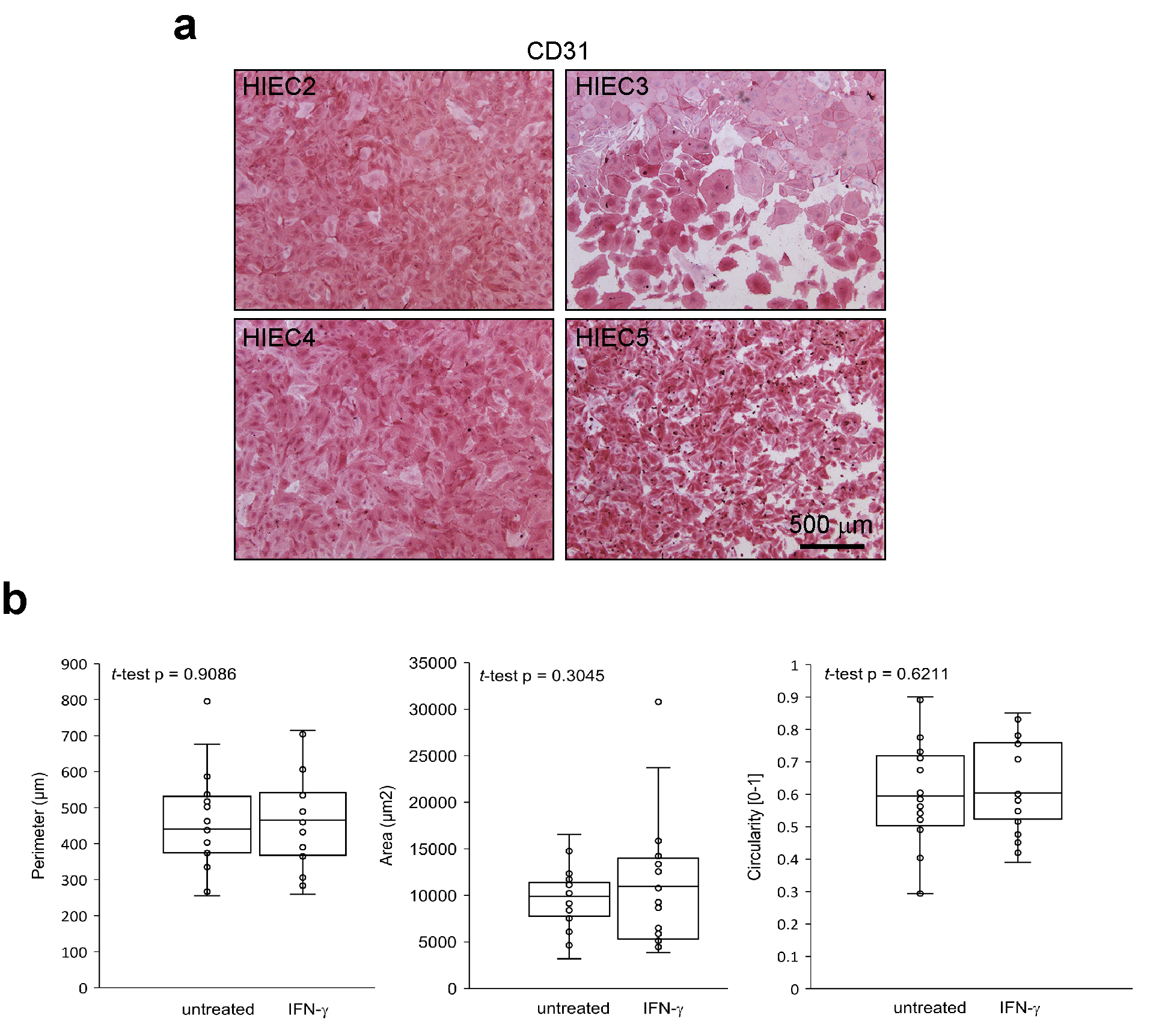

Supplement: Supplementary file 1 [file Image1.TIF]
