# Supplementary material for: Analysis of the interferon-γ-induced secretome of intestinal endothelial cells: putative impact on epithelial barrier dysfunction in IBD
Source: Front Cell Dev Biol. 2023 Aug 14;11:1213383. doi: 10.3389/fcell.2023.1213383 (PMC10460912; doi:10.3389/fcell.2023.1213383)
Supplement: Supplementary file 2 [file DataSheet1.docx]

**Supplemental methods and figure legend**

**Analysis of the interferon-γ induced secretome of intestinal endothelial cells: putative impact on epithelial barrier dysfunction in IBD**

Elisabeth Naschberger^1^, Christian Flierl^1^, Jinghao Huang^1^, Lena Erkert^2^, Reyes Gamez-Belmonte^2^, Miguel Gonzalez-Acera^2^, Magdalena Bober^3^, Martin Mehnert^3^, Christoph Becker^2^, Vera S. Schellerer^4^, Nathalie Britzen-Laurent^5,#^, Michael Stürzl^1,6,#^

^1^Division of Molecular and Experimental Surgery, Department of Surgery, Universitätsklinikum Erlangen, Friedrich-Alexander-Universität Erlangen-Nürnberg (FAU), Erlangen, Germany; ^2^Department of Medicine I, Universitätsklinikum Erlangen, Friedrich-Alexander-Universität Erlangen-Nürnberg (FAU), Erlangen, Germany; ^3^Biognosys AG, Schlieren, Switzerland; ^4^Department of Pediatric Surgery, University Medicine Greifswald, Greifswald, Germany. ^5^Division of Surgical Research, Department of Surgery, Universitätsklinikum Erlangen, Friedrich-Alexander-Universität Erlangen-Nürnberg (FAU), Erlangen, Germany; ^6^Comprehensive Cancer Center Erlangen-EMN, Universitätsklinikum, Erlangen, Erlangen, Germany

# these authors contributed equally

**Supplemental Methods**

**Patient recruitment, study approval.** All patients undergoing TEC isolation were diagnosed with CRC and did not receive pretreatment. Patients with IBD or known familial predisposition were excluded. Primary human intestinal endothelial cells (HIEC) were isolated from healthy colon areas of CRC patients (> 10 cm distance from primary tumor) according to a previously established protocol [1]. Tissue cut at a safety distance of at least 10 cm from the tumor is generally considered healthy and unaffected by the tumor [1, 2]. The procedure was approved by the local ethics committee (TuMiC study, No. 159_15B, Ethikkomission FAU Erlangen-Nürnberg) and all patients provided written informed consent.

**Isolation of HIEC.** HIEC were isolated from CRC patients according to a previously established protocol [3]. Cells were kept in EGM-2-MV medium (Lonza) at 37°C, 5% CO_2,_ were routinely tested for purity using CD31 immunocytochemistry and mycoplasma negativity using MycoAlert Mycoplasma Detection Kit (Lonza).

**Immunocytochemistry and immunohistochemistry.** CD31 immunocytochemistry was conducted as described [4].

**Isolation of RNA from HIEC.** RNA was isolated using RNeasy extraction kits (Qiagen) following the manufacturer´s instruction.

**RT-qPCR.** RT-qPCR for the targets GBP-1, CD31, vWF, CD105, VE-cadherin, CK-20, CD45, and desmin was performed as previously described with 20 ng total RNA used [4]. The primer/probe sequences for GBP-1 (gene ID, NCBI: 2633) were (all 5´-3´, forward/probe/reverse): CACCTTCGTGTACAATAGCATAGGA, AGGCTATGGACCAACTGTACTA, ATCGGATTCTATGTGTCAGCTCTGT.

**Cell stimulation**

In order to avoid unspecific cell lysis the stimulation with IFN-γ was carried out in the presence of low serum medium (0.5% FBS). We demonstrated previously that under these conditions the stimulation with IFN-γ in concentrations of 100 U/ml does not lead to a significant increase of cell lysis [5]. In order to further reduce putative unspecific lysis we used only 10 units/ml IFN-γ for stimulation in the present study. HIEC supernatants were harvested after 48 hrs of IFN-γ stimulation. We refrained from the use of synthetic media because these media are supplemented with human growth factors, cytokines and high concentrations of albumin. In contrast to the FBS proteins these supplemented human proteins cannot be distinguished from the secreted proteins by the MS analysis. Moreover, the software Spectronaut™ (Biognosys AG) was used for the mass spectrometry data analysis. This software includes the algorithm IDPicker [6] for protein inference which allows a clear discrimination in identification between proteins from different species based on the generated peptide spectrum matches. Therefore, the identification and quantification of proteins is not affected by the presence of proteins of a different species in a sample. In this study the mass spectrometry data were searched for human and bovine proteins. Proteins reported for human or bovine were identified with human or bovine specific peptides only.

**GBP-1-ELISA**. GBP-1-ELISA was performed as previously described [7]).

**Hyper reaction monitoring mass spectrometry (HRM^TM^ MS).** Unbiased proteomic profiling of secreted proteins from HIEC was performed with HRM^TM^ MS in cooperation with a commercial supplier (Biognosys, Zürich, Switzerland). Proteins in cell culture supernatants were digested with trypsin and subjected to HRM^TM^ MS. Sample-specific libraries were generated using direct search (directDIA^TM^). Exploratory data analysis included multivariate analysis and statistical testing to identify significantly regulated proteins. For testing of differential protein abundance, log2 peptide intensity ratios between replicate pairs for each protein were analyzed using a one sample t-test (μ = 0). P-values were corrected for overall FDR using the q-value approach [8].The following thresholds were applied for identification of significantly regulated proteins: q-value < 0.05; absolute average log2 ratio > 0.58 (fold-change > 1.5).

**Mouse models and gene expression analyses.** Mouse intestinal inflammation models were induced as described [9, 10]. RNA from the colonic samples were extracted using the peqGold total tissue RNA kit (peqlab GmbH) according to the manufacturer's instructions. Quality control, library preparation, and sequencing of the RNA samples were performed at Novogene (Cambridge, UK). Mapping, read quantification, differential expression and other graphic displays were done using publicly available and in-house bioinformatic tools.

**Statistics. qPCR, ELISA,** **area, perimeter and circularity** were statistically compared with unpaired double-tailed Student’*t*-test. *** = P <0.001, ** = P <0.01, and * = P <0.05.

**Protein secretome analysis**: tor testing of differential protein abundance, log2 peptide intensity ratios between replicate pairs for each protein were analyzed using a one sample t-test (μ = 0). P-values were corrected for overall FDR using the q-value approach [8].The following thresholds were applied for identification of significantly regulated proteins: q-value < 0.05; absolute average log2 ratio > 0.58 (fold-change > 1.5).

**Literature**

1. Schellerer, V.S., et al., *Endothelial cells of human colorectal cancer and healthy colon reveal phenotypic differences in culture.* Lab Invest, 2007. **87**(11): p. 1159-70.

2. *Evidenced-based Guideline for Colorectal Cancer.* 2019. **Version 2.1**.

3. Naschberger, E., et al., *Isolation of Human Endothelial Cells from Normal Colon and Colorectal Carcinoma - An Improved Protocol.* J Vis Exp, 2018(134).

4. Naschberger, E., et al., *Matricellular protein SPARCL1 regulates tumor microenvironment-dependent endothelial cell heterogeneity in colorectal carcinoma.* J Clin Invest, 2016. **126**(11): p. 4187-4204.

5. Naschberger, E., et al., *Human guanylate binding protein-1 is a secreted GTPase present in increased concentrations in the cerebrospinal fluid of patients with bacterial meningitis.* Am J Pathol, 2006. **169**(3): p. 1088-99.

6. Zhang, B., M.C. Chambers, and D.L. Tabb, *Proteomic parsimony through bipartite graph analysis improves accuracy and transparency.* J Proteome Res, 2007. **6**(9): p. 3549-57.

7. Naschberger, E., et al., *Processing and secretion of guanylate binding protein-1 depend on inflammatory caspase activity.* J Cell Mol Med, 2017. **21**(9): p. 1954-1966.

8. Storey, J.D. and R. Tibshirani, *Statistical significance for genomewide studies.* Proc Natl Acad Sci U S A, 2003. **100**(16): p. 9440-5.

9. Wirtz, S., et al., *Chemically induced mouse models of acute and chronic intestinal inflammation.* Nature Protocols, 2017. **12**(7): p. 1295-1309.

10. Eden, K., *Adoptive Transfer Colitis.* Methods Mol Biol, 2019. **1960**: p. 207-214.

**Supplemental figure 1:** **(a)** CD31 is uniformly expressed in all HIEC cultures used in this study. Scale bar corresponds to 500 µm. **(b)** Box blots showing size (area and perimeter) as well as the shape (circularity) of HIECs either untreated or treated with IFN-γ (n =20 per condition). Unpaired double-tailed Student’t-test was used for statistical comparison.
